# A Bespoke Electronic Health Journal for Monitoring Response to Botulinum Toxin in Treatment of Cervical Dystonia: Open-Label Observational Study of User Experience

**DOI:** 10.2196/45986

**Published:** 2023-08-23

**Authors:** Colin Edwards, Rebecca Borton, Anita Ross, Fiona Molloy

**Affiliations:** 1 patientMpower Ltd Dublin Ireland; 2 Beaumont Hospital Dublin Ireland

**Keywords:** cervical dystonia, electronic health journal, user experience, user acceptance testing, botulinum toxin, diary, acceptability, user testing, symptom control, spasm, muscle pain, spasmodic torticollis

## Abstract

**Background:**

The mainstay of treatment for cervical dystonia (CD) is regular botulinum toxin injections every 3-4 months. Clinical evaluation of response is dependent on the patient’s recall of how well symptoms responded to the previous injection. A mobile health app could assist both patients and health care professionals to monitor treatment benefits and side effects to assist with the selection of muscle and toxin dose to be injected at the next visit. The DystoniaDiary is a bespoke electronic health journal for monitoring symptoms of CD and response to treatment.

**Objective:**

The objective of this study was to assess the acceptability and utility of the DystoniaDiary in patients with CD treated with botulinum toxins as part of their usual care.

**Methods:**

In this open-label, single-center, single-arm observational study, patients attending a botulinum toxin injection clinic were invited to download the DystoniaDiary app. Patients selected up to 3 of their most troublesome CD symptoms (from a predefined list) and were prompted every 3 days to rate the control of these symptoms on a scale from 0 (very badly) to 100 (very well). Dates of onset and wearing off of response to injected botulinum toxin and responses to the Cervical Dystonia Impact Profile (CDIP-58) questionnaire at baseline and week 6 were also recorded in the app.

**Results:**

A total of 34 patients installed DystoniaDiary. Twenty-five patients (25/34, 74%) recorded data for ≥12 weeks and 21 patients (21/34, 62%) for ≥16 weeks. Median time between the first and last data input was 140 days with a median of 13 recordings per patient. User experience questionnaires at weeks 4 and 12 (20 respondents) indicated that the majority of respondents found the DystoniaDiary app easy to install and use, liked using it, would recommend it to others (19/20), and wished to continue using it (16/20). A smaller proportion indicated that the DystoniaDiary gave a greater sense of control in managing their CD (13/20). There was interindividual variation in patients’ perceptions of control of their symptoms after botulinum toxin injection. Response to treatment was apparent in the symptom control scores for some patients, whereas the severity of other patients’ symptoms did not appear to change after treatment.

**Conclusions:**

This observational study demonstrated that the DystoniaDiary app was perceived as useful and acceptable for a large proportion of this sample of patients with CD attending a botulinum toxin clinic. Patients with CD appear to be willing to regularly record symptom severity for at least the duration of a botulinum injection treatment cycle (12-16 weeks). This app may be useful in monitoring and optimizing individual patient responses to botulinum toxin injection.

## Introduction

Cervical dystonia (CD) is the most frequent form of focal dystonia and is characterized by involuntary contractions of specific neck muscles leading to abnormal movements of the head or unintentional adoption of sustained and frequently painful postures of the head, neck, and shoulders [1]. CD is a chronic, disabling, and often painful condition and there is no known cure.

Intramuscular botulinum toxin injections targeting the affected muscles are the mainstay of treatment for CD. This procedure is carried out by a physician with expertise in this field and knowledge of the anatomy of the neck region. Guidance for accurate selection of the muscles involved in the dystonic posturing using electromyography or visualization of the muscles using sonography is often used by the physician. Subjective benefit is usually reported 10-14 days postinjection. This improvement tends to plateau for a few months and a repeat injection is required every 3-4 months as the beneficial effects of treatment start to wane [2-4]. This is generally a well-tolerated treatment. Difficulty with swallowing may occur in a minority of patients but this is usually mild and resolves spontaneously within 7-10 days.

There are no objective markers available to assess both the symptom severity and response to treatment (unlike other chronic conditions such as hypertension or diabetes). Assessment of response to treatment is based predominantly on the patient’s subjective report as the symptoms and signs of CD have generally returned by the time of the follow-up botulinum toxin appointment. Patients may be asked to recall their best response over the time period since their last injection in order to inform the injector’s treatment decisions on dosing going forward. Doses are titrated based on this feedback over the course of several treatment cycles as every patient’s response is individual. Many patients require lifelong injections of botulinum toxin from the time of diagnosis.

Rating scales for the assessment of CD symptoms [1,5] have been developed but are complex to implement. For example, the Toronto Western Spasmodic Torticollis Rating Scale (TWISTRS) is a composite scale assessing physical findings, disability, and pain [1]. While assessment of outcomes of botulinum toxin therapy by validated methods such as TWISTRS are robust, they are complex and time-consuming for routine use in clinical practice [1] and are not usually applied in busy botulinum toxin clinics. Furthermore, these scales have not been rigorously tested for responsiveness to detect significant changes in clinical status after botulinum toxin injections and do not address the differentiation between head and neck CD subtypes. Furthermore, regardless of the rating scale applied, there are inherent difficulties in measuring the severity of CD due to its variability depending on fatigue, emotional stress, or activity.

In many countries, access to botulinum toxin clinics is suboptimal, and there are often long waiting times due to a shortage of trained injectors and high numbers of patients requiring treatment. Consequently, the clinics can be overextended and time is at a premium.

In general, botulinum toxin injections for CD are scheduled at fixed intervals irrespective of the duration of response to treatment. This leads to the possibility that patients may not have their botulinum toxin dose effectively titrated or response optimized.

We hypothesized that an electronic patient journal (app) to enable patients with CD to record the severity of their individual symptoms and their response to botulinum toxin over the period between injections would be useful and could enrich the discussion between patient and treating physician about their response to treatment at their next clinic visit.

A bespoke electronic health journal (DystoniaDiary) for patients with CD was developed and evaluated in this user acceptance testing (UAT) study. The primary objective was to assess the acceptability, utility, and ongoing compliance with the DystoniaDiary mobile app by patients with CD who are receiving treatment with injected botulinum toxins. A secondary objective was to assess the suitability of the data collected in the app to support discussions between patients and their health care professionals (HCPs) immediately prior to their next botulinum toxin injection.

The real-time data accrued by the DystoniaDiary app could lead to the development of a complete and comprehensive scale that could both classify the condition and accurately rate response to treatment.

## Methods

### Overview

This was a prospective, open-label, single-center, single-arm observational study of user experience of the DystoniaDiary electronic patient journal. The duration of observation per patient was planned to be at least one treatment cycle (expected to be 12 to 16 weeks).

No formal estimation of sample size was conducted for this pilot-scale study. Published estimates of appropriate sample size for UAT studies range from a sample size of 5 (for qualitative research) [6] to 20-40 users (for quantitative research) [7]. An enrollment target of 15-60 patient-users was set (based on expected recruitment within the timeframe of the study).

**Figure 1:**
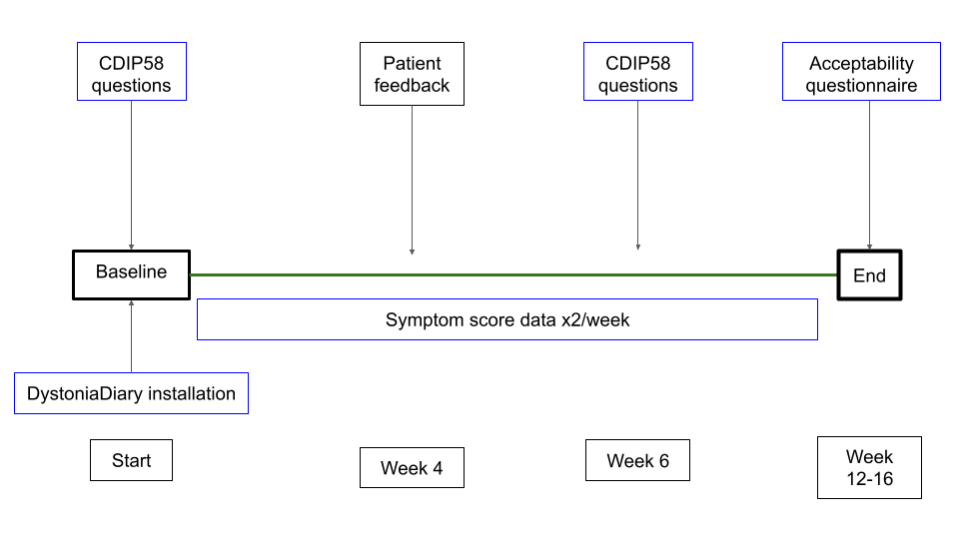
Design concept. The date of DystoniaDiary installation (start date) was intended to be the same as the date of the most recent botulinum injection. Patients were prompted to record control of their 3 most troublesome symptoms every 3 days and were asked to provide responses to the Cervical Dystonia Impact Profile (CDIP-58) questionnaire at baseline and at week 6. User experience questionnaires were sought at week 4 and at the end of the observation.

The study design is shown in Figure 1. All patients were assessed by a neurologist, and a diagnosis of CD was confirmed before being considered for inclusion. Patients attending the botulinum toxin clinic at the study center were invited to participate. There were no changes to the patients’ usual care during the study.

The DystoniaDiary electronic patient journal for CD was developed by patientMpower Ltd and commissioned by Ipsen Pharmaceuticals Ltd. It is available for download on the iOS App Store and Google Play Store platforms. A passcode is required to access the app after download.

The DystoniaDiary electronic patient journal was installed by the participating patients as an app on their own smartphone or tablet device. Access to the DystoniaDiary app was managed by a center-specific passcode provided by the HCP to all participants. Patients were only provided with the passcode after they had given informed consent to participate in the study. At the installation of the app, patients were asked to enter the passcode appropriate to that center.

**Figure 2:**
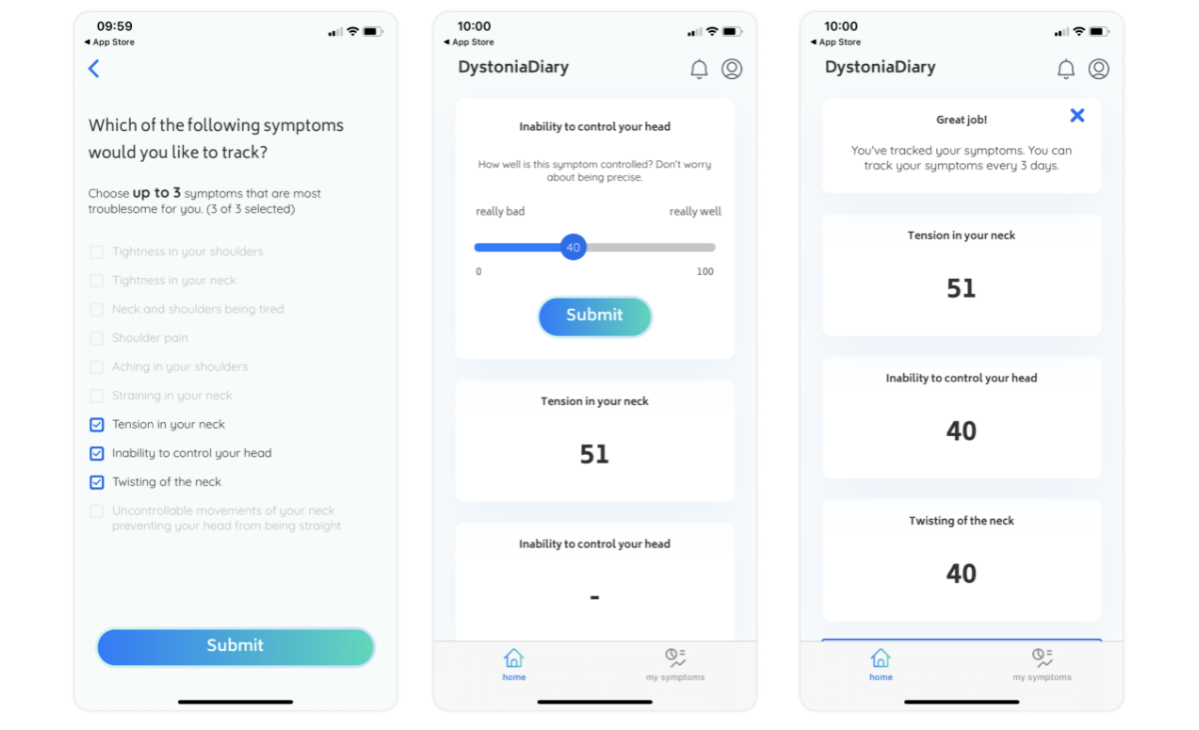
Example screenshot—symptom tracking. At onboarding, patients selected up to 3 of their most troublesome symptoms and rated the degree of symptom control on a scale ranging from 0 (very badly) to 100 (very well). Patients were prompted to record symptom control every 3 days during the observation period.

**Figure 3:**
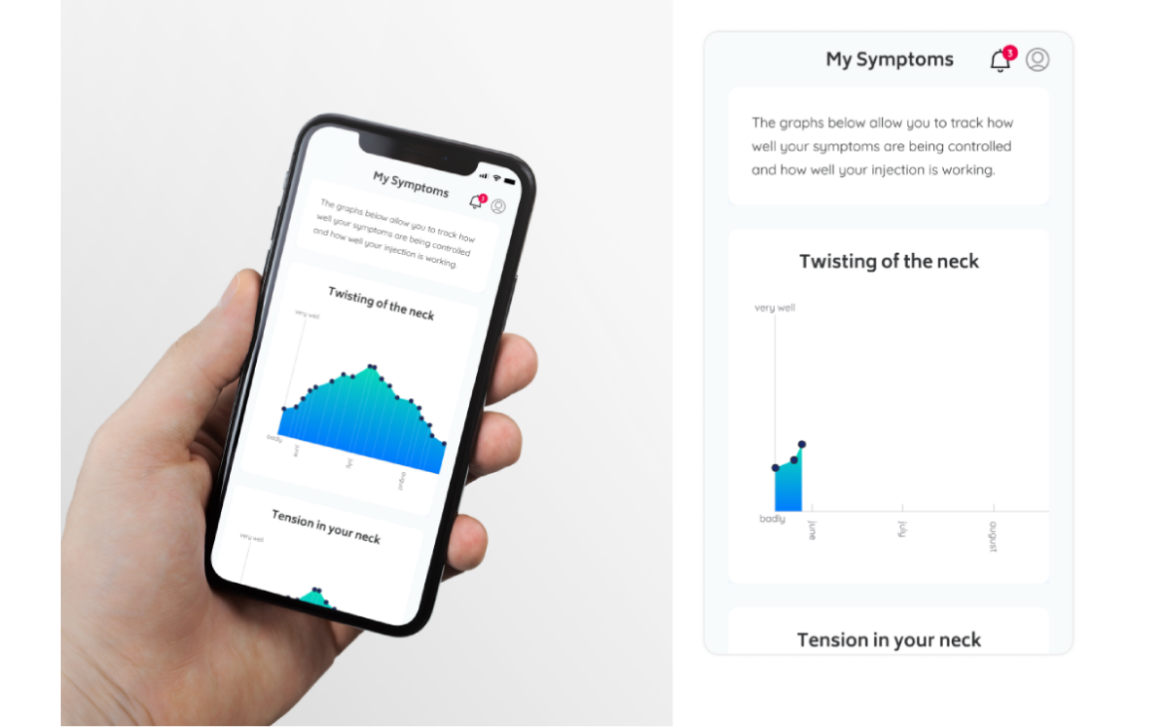
Example screenshot—symptom tracking over time. This example shows the patient’s view of the record of control of a single symptom (twisting of the neck). The left-hand panel shows the appearance after recording symptom control on 19 occasions over a 3-month period. In this example, symptom control improved until approximately 6 weeks after the last injection and then declined. The right-hand panel shows the appearance after recording symptom control on 3 occasions.

At installation of the DysoniaDiary app and after input of demographic data, patients selected up to 3 of their most troublesome symptoms (from a predefined list of 10 symptoms associated with CD; below) and then rated the extent of control of each of these symptoms on a sliding scale ranging from 0 (very badly) to 100 (very well). See Figure 2 for an example screenshot. Patients were asked to start recording the rating of symptom control as soon as possible after the date of their botulinum toxin injection. Patients were prompted every 3 days to repeat the rating of symptom control for the duration of the study and record this data within the app. An example of the patient’s view of the symptom control over time is shown in Figure 3.

The predefined list of 10 possible CD symptoms was as follows: “uncontrollable movements of your neck preventing your head from being straight, twisting of the neck, inability to control your head, tension in your neck, stiffness in your neck, aching in your shoulders, shoulder pain, neck and shoulders being tired, tightness in your neck and tightness in your shoulders.” The choice of the most troublesome symptoms could vary by person and patients did not necessarily experience all of the symptoms in the predefined list. For example, some patients may have experienced neck stiffness, whereas others may have experienced inability to control their head as their worst symptoms.

**Figure 4:**
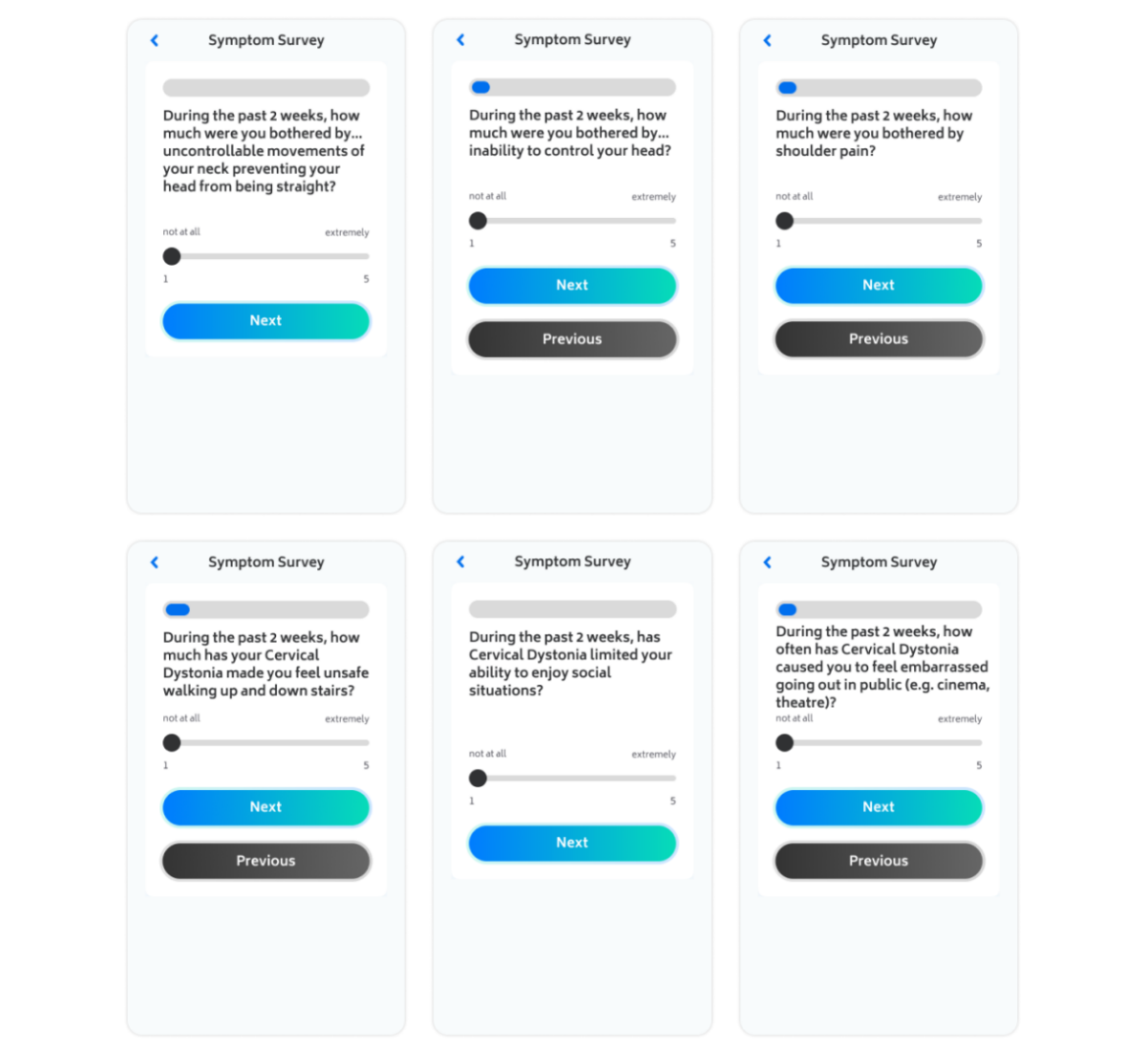
Example screenshot—CDIP-58 tracking at week 6. At baseline and week 6, patients recorded responses to CDIP-58 questions on the impact of cervical dystonia on their life in the 2 weeks prior to the date of response. CDIP-58: Cervical Dystonia Impact Profile.

Patients were also asked to record symptom severity in the app using domains derived from the Cervical Dystonia Impact Profile (CDIP-58) questionnaire [8] at baseline and at week 6 of the observation period. The CDIP-58 questions are shown in Multimedia Appendix 1 and an example screenshot is shown in Figure 4. For questions 1-13, 18, and 19, responses were captured on a sliding scale ranging from 0 (not at all) to 100 (extremely). For questions 14-17 and 20, responses were captured on a sliding scale ranging from 0 (none of the time) to 100 (all of the time). The CDIP-58 questionnaire captures the patient experience of the impact of CD on their life in the 2 weeks prior to the date of response.

Patients were asked to record in the app the date they first noted the benefit from their botulinum toxin injection, the date benefit started to wear off and the date benefit completely wore off (if applicable) and their satisfaction with this injection compared with their last injection (on a scale from 0, not satisfied to 5, very satisfied).

At weeks 4 and 12, patients completed user experience surveys to provide feedback on their experience of using the app. The survey questions are in Multimedia Appendix 2.

### Ethics Approval

The study was approved by the Research Ethics Committee (REC) at Beaumont Hospital Dublin on 25 May 2021 (REC reference 21/27) prior to starting. The study procedures were discussed with potential participants attending the botulinum toxin clinic and all patients gave written informed consent prior to participation. Patients did not receive any compensation for taking part. The first patient was enrolled in August 2021 and data up to April 30, 2022, are included in this analysis.

## Results

Thirty-four patients installed the DystoniaDiary app and added data on at least one occasion. Data were recorded for ≥12 weeks by 25 patients (25/34, 74%) and for ≥16 weeks by 21 patients (21/34, 62%). Baseline demographics are shown in Table 1. There was a high proportion of female patients (25/34, 74%) in the study population. The majority of patients had an established diagnosis of CD prior to entry with a median time since the first ever botulinum toxin injection of 9.33 years. The interval between patient installation of the DystoniaDiary app and their most recent botulinum toxin injection was short (median 4 days) and 32 of 34 patients (94%) started to enter data on the day of app installation. During the timeframe of the study, 31 of 34 patients (91%) received a second botulinum toxin injection with a median interval between injections of 112 days. Data were recorded by 21 patients (21/34, 62%) after the date of their second injection.

At baseline, 30 patients (30/34, 88%) recorded data on 3 symptoms and 4 patients (4/34, 12%) on 2 symptoms. The most common reported CD symptom was “tension in your neck” (17/34, 50%) followed by “tightness in your neck,” “straining (stiffness) in your neck” (14/34, 41%), and “inability to control your head” (12/34, 35%).

**Figure 5:**
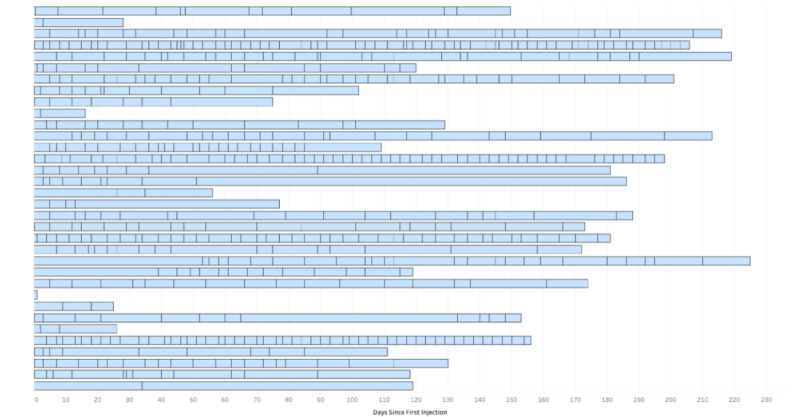
Duration versus number of data entry days. Heatmap showing the duration and number of occasions of data entry by patients. Each horizontal bar represents the duration of the recording of data by each individual patient after installation of the DystoniaDiary app. The vertical lines within each bar indicate a day on which the patient entered control symptom score data on the app.

The duration and frequency of use of the app was variable. All patients recorded ratings of symptom control at least once. The median interval between first and last data input was 140 days and the median number of occasions where symptom score data were entered was 13. The median number of occasions where symptom score data were entered per week for each patient was 0.81 per week which was less than planned (2 per week). The duration and frequency of input of symptom control score data is illustrated in Figure 5 and summarized in Table 2. Some patients entered data very frequently throughout the observation period.

CDIP-58 data were entered by 23 patients (23/34, 68%) at median 45 days after baseline. Information on treatment effectiveness was entered by 23 patients (23/34, 68%) at median 40 days after baseline with 20 of 23 patients reporting benefit from their botulinum toxin injection on or before that date.

User experience surveys were provided by 15 patients (15/34, 44%) at week 4 and by 20 patients (20/34, 59%) at week 12. At week 4, 1 respondent reported that installation of the DystoniaDiary app was “difficult” and that the instructions for installation were “a bit unclear.” All other respondents (14/15) reported that installation was “easy” or “very easy” and that the instructions were “very clear” or “clear.”

At week 12, the majority of respondents reported that recording information on the DystoniaDiary was “very easy” or “easy” (17/20). On a scale of 1 (confusing) to 4 (very clear), 12/20 respondents rated their experience of using the DystoniaDiary app as “4 (very clear).” The majority of respondents reported positive responses to other questions (summarized in Table 3) with 16/20 respondents expressing a wish to continue using the app after the end of the study.

**Table 1:** Baseline demographics^a^.

Data	Median (IQR)	Mean (SD)	Range
Age (years)	—	54 (12)	21-82
Time since first ever botulinum toxin injection^b^ (years)	9.33 (3.5-13.5)	—^c^	0-34.25
Interval between DystoniaDiary installation and most recent botulinum toxin injection (days)	4 (1-6)	—	0-54
Interval betweem second injection and most recent botulinum toxin^d^ (days)	112 (110-129)	—	59-191

^a^Data not available for 1 patient.

^b^Patient-reported date of first-ever botulinum toxin injection.

^c^Not available.

^d^A total of 91% of patients (31/34) received a second botulinum toxin injection during the timeframe of the study.

**Table 2:** Time intervals between data input and survey responses.

	Median (IQR)	Mean (SD)	Patients (N=34), n (% of total)
Last-first data input interval, (days)	140 (81-180)	127 (65)	34 (100)
Number of times symptom control score data were input, (n)	13 (8-25)	18 (16)	34 (100)
Number of times symptom control score data were input per week for each patient, (n)	0.81 (0.69-1.14)	0.97 (0.52)	34 (100)
Interval between input of first CDIP-58 data and baseline, (days)	45 (39-61)	52 (25)	23 (68)
Interval between 4-week patient opinion survey and baseline, (days)	29 (28-33)	31 (4)	15 (44)
Interval between 12-week patient opinion survey and baseline, (days)	84 (83-85)	85 (2)	20 (59)
Interval between treatment effectiveness information input and baseline, (days)	40 (32-59)	50 (25)	23 (68)

**Table 3:** User survey responses at week 12.

Survey question	Responses, n	
	Yes	No
I like using the DystoniaDiary application.	19	1
Using the DystoniaDiary application gave me a greater sense of control in managing my cervical dystonia.	13	7
Do you want to continue using the DystoniaDiary after the end of the project?	16	4
Would you recommend the DystoniaDiary for other people with cervical dystonia?	19	1
In your opinion, does the app work well?	18^a^	2^b^

^a^The response to this question was “well.”

^b^The response to this question was “not so well.”

## Discussion

This is the first evaluation of the DystoniaDiary app, which was specifically developed for patients with CD and facilitates them in monitoring their most troublesome symptoms.

This observational study indicated that in this patient population, most patient-users found the app to be useful and easy to use and they were willing to use it frequently for prolonged periods of time.

**Figure 6:**
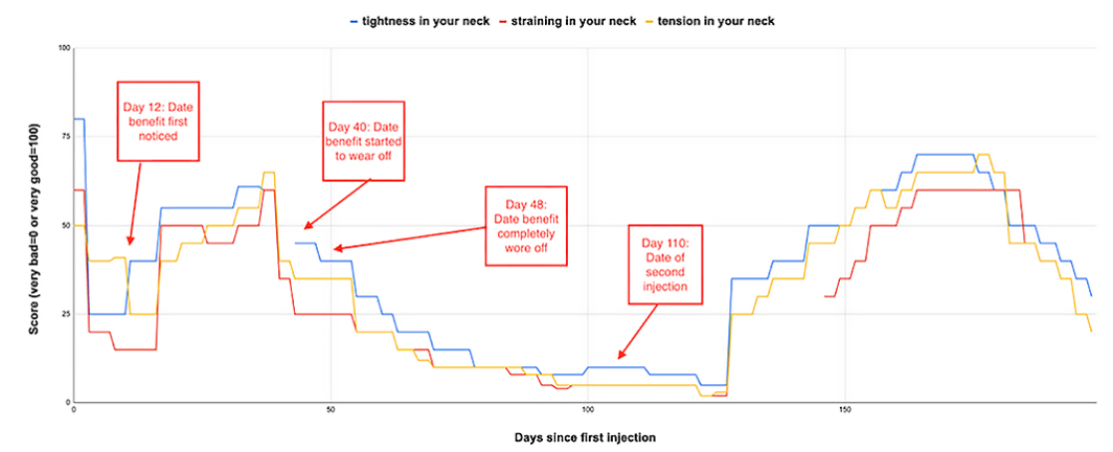
Example patient profile 1. This patient's ratings of control of the following symptoms over time are shown: tightness in the neck (blue line), straining in the neck (red line), and tension in the neck (yellow line). Day 0 is the date of the botulinum toxin injection immediately prior to installation of DystoniaDiary (x-axis). A high score (max 100) indicates good control of symptoms (y-axis). The time points indicated are date treatment benefit first noted, benefit starting to wear off, benefit completely worn off, and date of second botulinum toxin.

**Figure 7:**
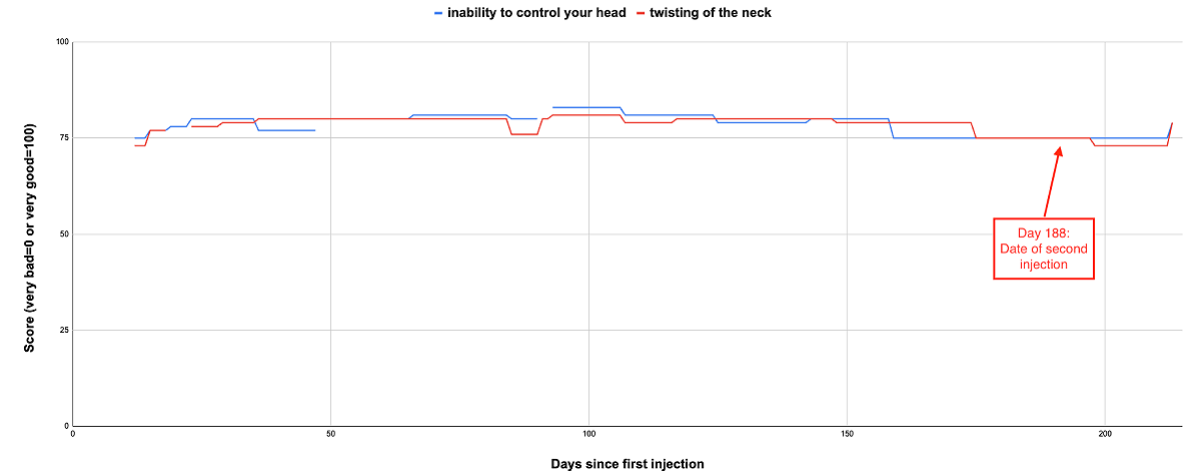
Example patient profile 2. This patient's ratings of control of the following symptoms over time are shown: inability to control the head (blue line) and twisting of the straining in the neck (red line). Day 0 is the date of the botulinum toxin injection immediately prior to installation of DystoniaDiary (x-axis). A high score (max 100) indicates good control of symptoms (y-axis). The date of the second botulinum toxin is indicated.

The multiple data points on patient-rated control of symptoms over time can be visualized to provide a long-term view of response to injected botulinum toxin treatment and could support clinical decisions on the timing or dosing of subsequent therapy for an individual patient. An example of a single patient who recorded data over a 197-day period is shown in Figure 6. In this case, the patient reported the onset of the benefit of treatment at 12 days after injection, with the wearing off of benefit starting at day 40 and loss of benefit at day 48. The patient-reported control of their symptom scores varies over time and there is an apparent response in symptom control after their initial botulinum toxin injection and after a second injection at day 110. Similar patterns of change in control of symptom scores relative to dates of injection were observed in 6 other patients with long-term data. In contrast, some patients recorded very little variation in control of symptom scores over time (observed in 12 patients with long-term data). An example is shown in Figure 7.

Patient rating of their symptom control or severity may be very subjective. It appears that some patients (as in the example in Figure 7) may perceive their condition as always being the same irrespective of any treatment. It is not clear if this reflects a high degree of tolerance of their symptoms by the patient or reflects a true lack of effect of the injected treatment. An alternative explanation is that the DystoniaDiary was not a suitable instrument to detect symptom control for this patient. Other patients (as in the example in Figure 6) perceive their condition as changing over time in response to treatments, suggesting that at least in some cases, this approach could be used to monitor treatment response over time.

A limitation of the study is that patients were not assessed with a validated assessment tool (eg, TWISTRS) [1] at baseline or at follow-up visits, so an objective assessment of their response to toxin treatment was not available. A comparison between validated measures of treatment response (eg, TWISTRS) and the subjective symptom control scores reported in DystoniaDiary would have strengthened this study. The observation that some patients did not report change in symptom control (as in the example in Figure 7) is in contrast to previous experience at this treatment center, where over 50% of patients report improvement on a visual analog scale after toxin injection.

There was some overlap in the terms used in DystoniaDiary to report symptoms (eg, “tension” vs “tightness” vs “straining [stiffness]”) affecting the neck or shoulder. These terms were derived from the CDIP-58 questionnaire [7]. It may be more useful to have a single phrase (eg, “tightness in the neck”) describing this type of symptom in future iterations of the app.

The usefulness of the DystoniaDiary app would be improved if the patient-recorded information was made available to their clinical care centers in real time (via a browser-based portal) to monitor treatment effectiveness and potentially adjust treatment schedules. This approach has been widely adopted in monitoring respiratory diseases, including COVID-19 [9], interstitial lung disease [10], and post–lung transplantation care [11].

At the time of writing, another electronic health diary (MyDystonia) is available [12]. This app enables patients to record symptoms over time and schedule medical appointments. It is designed for multiple types of dystonia rather than specifically for CD (unlike DystoniaDiary) and does not appear to track treatment dates and treatment effectiveness. To date, there do not appear to be any publications describing the use of the MyDystonia app or any other app designed specifically for patients with CD.

In conclusion, this observational study demonstrated that the DystoniaDiary app was useful and acceptable for a large proportion of this sample of patients with CD attending a botulinum toxin clinic. Patients appear to be willing to regularly record symptom severity for at least the duration of a botulinum injection treatment cycle (12-16 weeks). Response to treatment was apparent in some patients, whereas the severity of other patients’ symptoms did not appear to change despite treatment. This app may be useful in monitoring and optimizing individual patient responses to botulinum toxin.

## Appendix

Multimedia Appendix 1 Cervical Dystonia Impact Profile (CDIP-58) questions.

Multimedia Appendix 2 Survey questions at week 4 and week 12.

## Abbreviations

CD: cervical dystonia
CDIP-58: Cervical Dystonia Impact Profile
HCP: health care professional
REC: Research Ethics Committee
TWSTRS: Toronto Western Spasmodic Torticollis Rating Scale
UAT: user acceptance testing

## Data Availability

The anonymized data sets generated or analyzed during this study are available from the corresponding author upon reasonable request.
