# Supplementary material for: A Bespoke Electronic Health Journal for Monitoring Response to Botulinum Toxin in Treatment of Cervical Dystonia: Open-Label Observational Study of User Experience
Source: JMIR Form Res. 2023 Aug 23;7:e45986. doi: 10.2196/45986 (PMC10483297; doi:10.2196/45986)
Supplement: Multimedia Appendix 1 [file formative_v7i1e45986_app1.docx]

# Multimedia Appendix 1

## CDIP-58 questions

Patients were asked to provide responses to the following questions based on the validated CDIP-58 questionnaire at baseline and at Week 6. For questions 1-14, 18, and 19, responses were captured on a sliding scale ranging from 0 (not at all) to 100 (extremely). For questions 15-17 and 20, responses were captured on a sliding scale ranging from 0 (none of the time) to 100 (all of the time).

1. During the past 2 weeks how much were you bothered by uncontrollable movements of your neck preventing your head from being straight?
2. During the past 2 weeks how much were you bothered by twisting of the neck?
3. During the past 2 weeks how much were you bothered by inability to control your head?
4. During the past 2 weeks how much were you bothered by tension in your neck?
5. During the past 2 weeks how much were you bothered by stiffness in your neck?
6. During the past 2 weeks how much were you bothered by shoulder pain?
7. During the past 2 weeks how much were you bothered by neck and shoulders being tired?
8. During the past 2 weeks how much were you bothered by tightness in your neck?
9. During the past 2 weeks how much were you bothered by tightness in your shoulders?
10. During the past 2 weeks, has cervical dystonia limited your ability to carry out your usual daily activities (eg, cleaning, cooking, and work)?
11. During the past 2 weeks, how much has your cervical dystonia: limited how far you are able to walk?
12. During the past 2 weeks, how much has your cervical dystonia increased the effort needed for you to walk?
13. During the past 2 weeks, how much has your cervical dystonia made you feel unsafe walking up and down stairs?
14. During the past 2 weeks, how often did you have trouble falling asleep because of the symptoms of your cervical dystonia?
15. During the past 2 weeks, how often did you have a restless sleep because of the symptoms of your cervical dystonia?
16. During the past 2 weeks, how often did you wake up because of the symptoms of your cervical dystonia?
17. During the past 2 weeks, how often did you not get the amount of sleep that you needed because of the symptoms of your cervical dystonia?
18. During the past 2 weeks, has cervical dystonia limited your ability to enjoy social situations?
19. During the past 2 weeks, has cervical dystonia limited your ability to socialize with friends or family?
20. During the past 2 weeks, how often has cervical dystonia caused you to feel embarrassed going out in public (eg, cinema, theatre)?
