# Supplementary material for: A Bespoke Electronic Health Journal for Monitoring Response to Botulinum Toxin in Treatment of Cervical Dystonia: Open-Label Observational Study of User Experience
Source: JMIR Form Res. 2023 Aug 23;7:e45986. doi: 10.2196/45986 (PMC10483297; doi:10.2196/45986)
Supplement: Multimedia Appendix 2 [file formative_v7i1e45986_app2.docx]

## Supplement 2

**Survey questions at week 4 and week 12:**

I like using the DystoniaDiary application

yes/no

Using the DystoniaDiary application gave me a greater sense of control in managing my cervical dystonia

yes/no

Rate the difficulty of recording information on DystoniaDiary

very easy/easy/difficult/very difficult

Do you want to continue using the DystoniaDiary after the end of the project?

yes/no. If no, why not

Would you recommend the DystoniaDiary for other people with dystonia?

yes/no

What other measurements, reminders, or information would be useful to have on in the DystoniaDiary?

free text

In your opinion does the app work well?

well/not so well

What would you change about this app to make it more user friendly and useful?

free text

Rate your experience with using this app

1 (confusing)/2/3/4 (very clear)

Describe the benefits or disadvantages of using the DystoniaDiary?

free text

**Additional survey questions at week 4:**

How clear were the instructions for setting up the DystoniaDiary application?

very clear/clear/a bit unclear/very unclear

Rate the difficulty of installing the DystoniaDiary?

very easy/easy/difficult/very difficult
